# Natural variation in *Beauty Mark* is associated with UV-based geographical adaptation in *Gossypium* species

**DOI:** 10.1186/s12915-023-01591-5

**Published:** 2023-05-12

**Authors:** Muhammad Ali Abid, Qi Zhou, Mubashir Abbas, Haiyan He, Zhigang Meng, Yuan Wang, Yunxiao Wei, Sandui Guo, Rui Zhang, Chengzhen Liang

**Affiliations:** Biotechnology Research Institute, Chinese Academy of Agricultural Sciences, Beijing, 100081 China

**Keywords:** *Gossypium*, Beauty Mark, UV light, Anthocyanin, Geographical adaptation

## Abstract

**Background:**

Anthocyanins, a class of specialized metabolites that are ubiquitous among plant species, have attracted a great deal of attention from plant biologists due to their chemical diversity. They confer purple, pink, and blue colors that attract pollinators, protect plants from ultraviolet (UV) radiation, and scavenge reactive oxygen species (ROS) to facilitate plant survival during abiotic stress. In a previous study, we identified *Beauty Mark* (*BM*) in *Gossypium barbadense* as an activator of the anthocyanin biosynthesis pathway; this gene also directly led to the formation of a pollinator-attracting purple spot.

**Results:**

Here, we found that a single nucleotide polymorphism (SNP) (C/T) within the *BM* coding sequence was responsible for variations in this trait. Transient expression assays of *BM* from *G. barbadense* and *G. hirsutum* in *Nicotiana benthamiana* using luciferase reporter gene also suggested that SNPs in the coding sequence could be responsible for the absent beauty mark phenotype observed in *G. hirsutum.* We next demonstrated that the beauty mark and UV floral patterns are associated phenotypes and that UV exposure resulted in increased ROS generation in floral tissues; BM thus contributed to ROS scavenging in *G. barbadense* and wild cotton plants with flowers containing the beauty mark. Furthermore, a nucleotide diversity analysis and Tajima’s *D* Test suggested that there have been strong selective sweeps in the *GhBM* locus during *G. hirsutum* domestication.

**Conclusions:**

Taken together, these results suggest that cotton species differ in their approaches to absorbing or reflecting UV light and thus exhibit variations in floral anthocyanin biosynthesis to scavenge reactive ROS; furthermore, these traits are related to the geographic distribution of cotton species.

**Supplementary Information:**

The online version contains supplementary material available at 10.1186/s12915-023-01591-5.

## Background

Pollinator-mediated selection is commonly associated with intraspecies variation in flower color, which can be a reliable signal of nectar content and thus directly impact pollinator visiting rates [1]. However, this mechanism cannot account for floral color differences in self-pollinating species. In such cases, indirect selection via biotic and abiotic stresses may be responsible for variations in flower color [2]. Abiotic factors such as temperature, drought stress, and exposure to ultraviolet (UV) radiation influence differences in flower color; pigmentation in flowers can confer fitness advantages under heat and drought stress [3–6]. Furthermore, compounds that yield floral pigmentation act as antioxidants, protecting against the reactive oxygen species (ROS) produced by UV-B exposure [7]. Such compounds include specialized metabolites such as flavonoids and anthocyanins, which are essential for plant survival under multiple environmental stresses [8]. Anthocyanins are ubiquitously distributed among members of the plant kingdom and have attracted the attention of plant biologists due to their chemical diversity and ecological significance [9, 10]. They endow plant organs with purple, pink, or blue colors, which attract pollinators and seed dispersers [11] and protect plants against UV radiation [12] and pathogen invasion [13]. In addition, antioxidant activity allows anthocyanins to scavenge ROS, facilitating plant survival in response to abiotic stresses [14]. Anthocyanin accumulation in plant organs is recognized as a visible biomarker to indicate past environmental stresses. Plants have developed sophisticated molecular mechanisms to synchronize anthocyanin biosynthesis with growth and development. However, the underlying molecular mechanism by which stressors induce anthocyanin biosynthesis is not clear.

In many plant species, anthocyanin biosynthesis and the associated regulatory mechanisms at the transcriptional level have been thoroughly elucidated [15, 16]. In higher plants, anthocyanin biosynthesis-related gene expression is regulated by the conserved MBW core activation complex, which includes the v-myb avian myeloblastosis viral oncogene homolog (MYB), basic helix-loop-helix (bHLH), and WD40 subunits. R2R3 MYB regulators have been demonstrated to be transcriptional activators of the anthocyanin biosynthetic pathway in many species, such as *Arabidopsis* [17], petunia [18], tomato [19], grapevine [20], maize [21], potato [22], sweet potato [23], and apples [24]. Some MYB transcription factors (TFs) have also been identified as repressors of the anthocyanin biosynthetic pathway, including *MYB1* in strawberries [25], *MYB308* and *MYB330* in snapdragon [26], MYB10 in apples [27], MYB4a and MYB4b in grapes [28], MYB4 in *Arabidopsis* [29], MYB27 in petunia [30], and MYBF2 in ginkgo [31]. Petals with large spots and discrete anthocyanin depositions that contrast with the background flower coloration occur in members of the families *Liliaceae*, *Orchidaceae*, *Asteraceae*, *Papaveraceae*, *Fabaceae*, *Malvaceae*, and many others, indicating that this trait has evolved multiple times independently. Numerous field studies have revealed that petal spots are important in mediating interactions with pollinators [1, 32, 33].

Increased plant exposure to UV-B radiation can increase the concentrations of anthocyanins and other specialized metabolites associated with floral pigmentation in both the visible and UV spectra [34]. Biosynthesis of UV light-absorbing compounds (flavonols) may attenuate damage caused by excess irradiance [7, 35]. There is substantial empirical evidence that floral anthocyanins can shield chloroplasts from UV damage [12, 36] by capturing a portion of supernumerary photons that would otherwise strike the chloroplasts, increasing ROS production and therefore ROS-triggered damage [37]. Previous studies in model species such as petunia and snapdragon have clarified the genetic control of some pigment patterns (and the association of such pigments with UV absorbance) [38] and of variation in pigment intensity in different regions of the corolla [30]. Cultivated upland cotton lacks an area of anthocyanin pigmentation at the base (known as a petal spot), but such spots are not uncommon in the so-called primitive cottons or race stocks [39]. For example, *G. barbadense* and *G. arboreum* (Asiatic cotton) have petal spots. This characteristic has been identified as a marker and is used by breeders and seed producers [40]. However, general understandings of cotton petal spot development and regulation at the molecular level remain poor.

Recently, we cloned *Beauty Mark* (*BM*) from *G. barbadense* using a map-based cloning method. We demonstrated that *GbBM* activated anthocyanin production in the petal spot region and directly led to the formation of a pollinator-attracting purple spot (the beauty mark) at the base of flower petals. We confirmed that the presence of these spots significantly increased the number of visiting honeybees, and that introducing the *GbBM* allele from *G. barbadense* to *G. hirsutum* improved pollination [41]. In the present study, we found the causal single nucleotide polymorphism (SNP) within the *GbBM* coding sequence (CDS) that was responsible for beauty mark development; furthermore, the beauty mark was shown to be associated with UV floral patterning. We demonstrated that the co-occurrence of the beauty mark and UV floral patterning protected flower reproductive tissues from UV light by producing antioxidants. Finally, we demonstrated the role of geographic distribution and associated UV light intensity in cotton species evolution.

## Results

### Co-occurrence of the beauty mark phenotype and UV patterns in *Gossypium* species

**Fig. 1:**
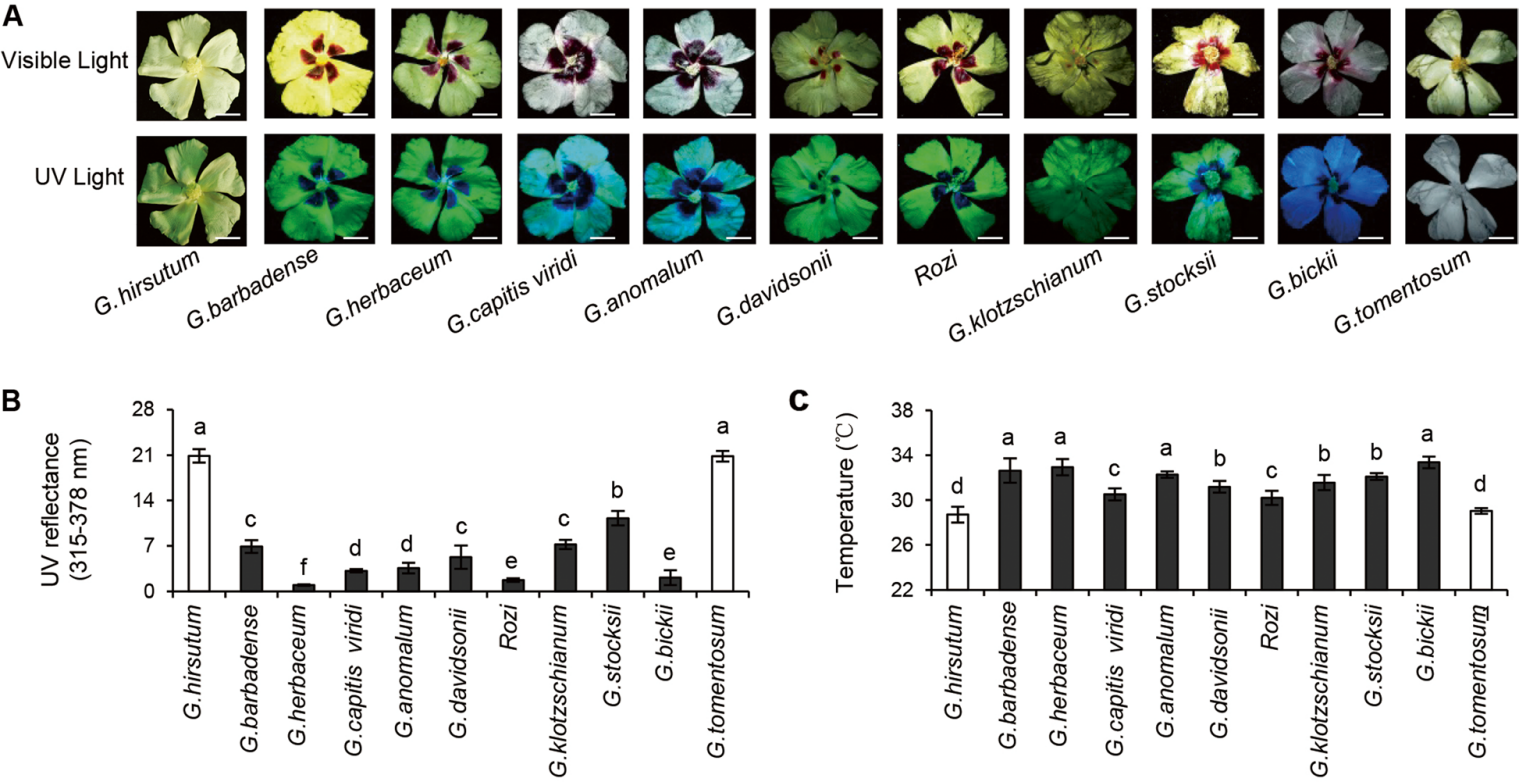
Effects of co-occurrence of Beauty Mark phenotype and UV light pattern on floral petals of *Gossypium* species. **A** Flowers of *Gossypium* species under visible light and UV light. Scale bar, 2 cm. The data were obtained from three independent replicates. **B** UV-reflectance data measured from floral petal of *Gossypium* species. **C** Temperature data measured from floral petal of *Gossypium* species. The data in **B** and **C** were analyzed by ANOVA one-way comparison followed by LSD test. Different letters above the bars indicate a significant difference at *P* < 0.05

We previously studied phenotypic differences between *G. barbadense* and *G. hirsutum* flowers to determine the genuine signals in a signaling relationship between honeybee pollinators and flowers. A dark purple mark at the base of the petals is characteristic of *G. barbadense* genotypes, whereas most of the cultivated *G. hirsutum* genotypes do not have a beauty mark. We here studied beauty marks in cultivated cotton *G. herbaceum*, and wild cotton species, namely *G. capitis-viridis*, *G. anomalum*, *G. davidsonii*, Rozi, *G. klotzschianum*, *G. stocksii*, and *G. bickii*, which had beauty marks at the petal bases of petals. We also studied one species, *G. tomentosum*, which did not have a beauty mark. The intensity and size of the beauty marks varied between species. Beauty marks and petal UV patterns were previously found to be the most conspicuous traits associated with honeybee visits. We therefore focused on the association between anthocyanin pigmentation in the beauty mark and UV-responsive flavonol compounds. When exposed to UV light, the petals of *G. barbadense*, *G. herbaceum*, *G. capitis-viridis*, *G. anomalum*, *G. davidsonii*, Rozi, *G. klotzschianum*, *G. stocksii*, and *G. bickii* significantly darkened, whereas the non-beauty-marked petals of *G. hirsutum* and *G. tomentosum* remained relatively bright (Fig. 1A). Consistent with those data, the UV light patterns of the petals differed between species with and without beauty marks (Fig. 1B). UV patterning may lead to an increase in petal temperature in response to UV exposure. We therefore proposed that UV light exposure may lead to differences in temperature and metabolic activities in floral tissues of *Gossypium* species with and without beauty marks. To test this hypothesis, we measured the petal temperatures of wild and cultivated *Gossypium* species. The temperatures of *G. barbadense* and wild cotton petals that contained the beauty mark were ~ 2 °C higher than those of *G. hirsutum* and *G. tomentosum* (Fig. 1C).

### *Beauty Mark* decreased cellular H2O2 accumulation in *Gossypium* petals

**Fig. 2:**
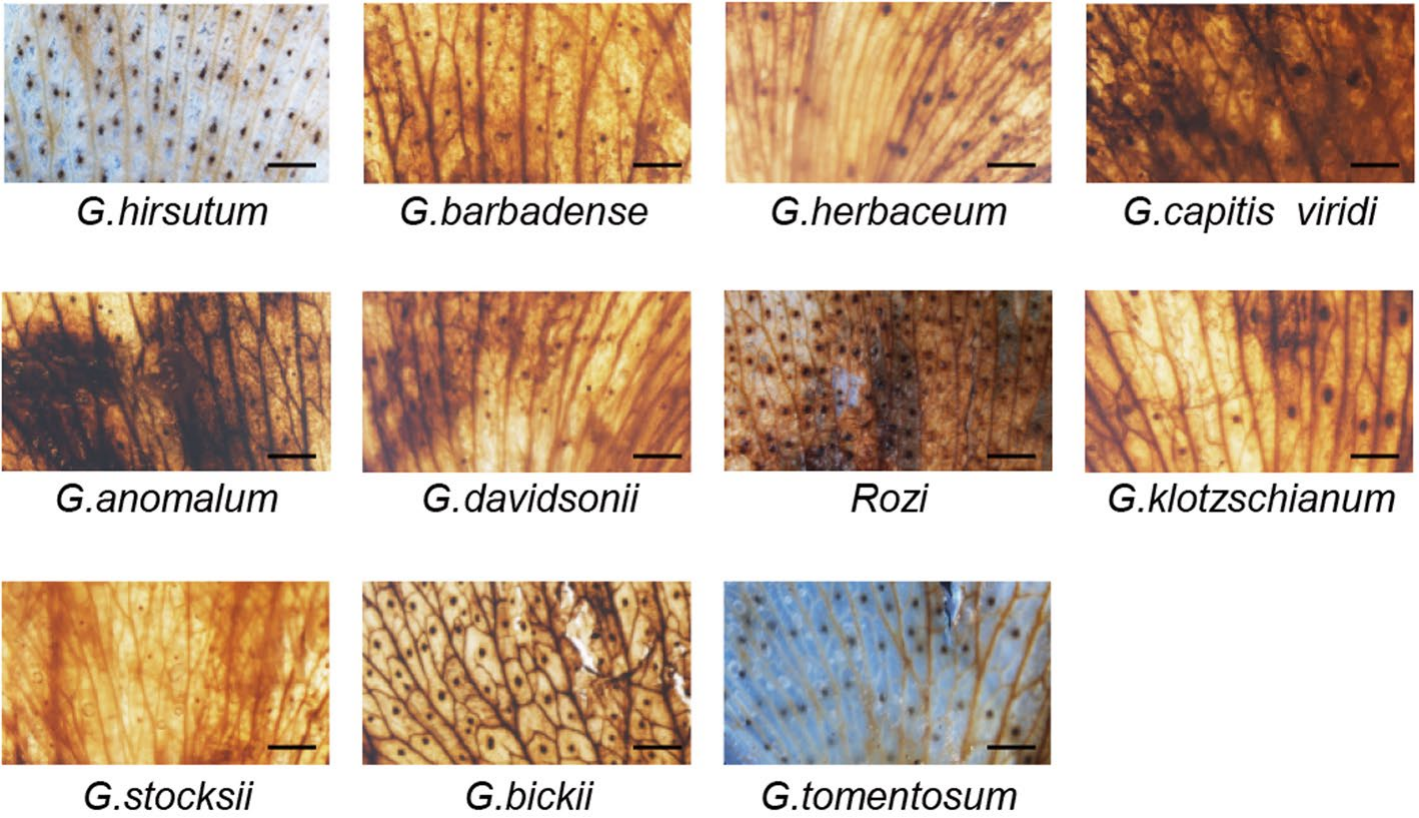
Staining of floral petals of *Gossypium* species with DAB to determine the contents of ROS produced. Scale bar, 3 mm. The data were obtained from three independent replicates

Anthocyanins are more efficient than ascorbate and tocopherol at scavenging nearly all types of ROS and free radicals [42]. Increased temperatures generate ROS and free radicals. To cope with these challenges, cotton plants may increase anthocyanin biosynthesis to scavenge ROS. We therefore used 3,3′-diaminobenzidine (DAB) staining to measure ROS (H_2_O_2_) in the petals of *Gossypium* species with and without beauty marks. Strikingly, the petals of beauty mark-containing *Gossypium* species (both in the beauty mark and non-beauty mark regions) showed significant dark DAB staining. In comparison, the petals of *G. hirsutum* and *G. tomentosum* had reduced H_2_O_2_ levels (Fig. 2).

**Fig. 3:**
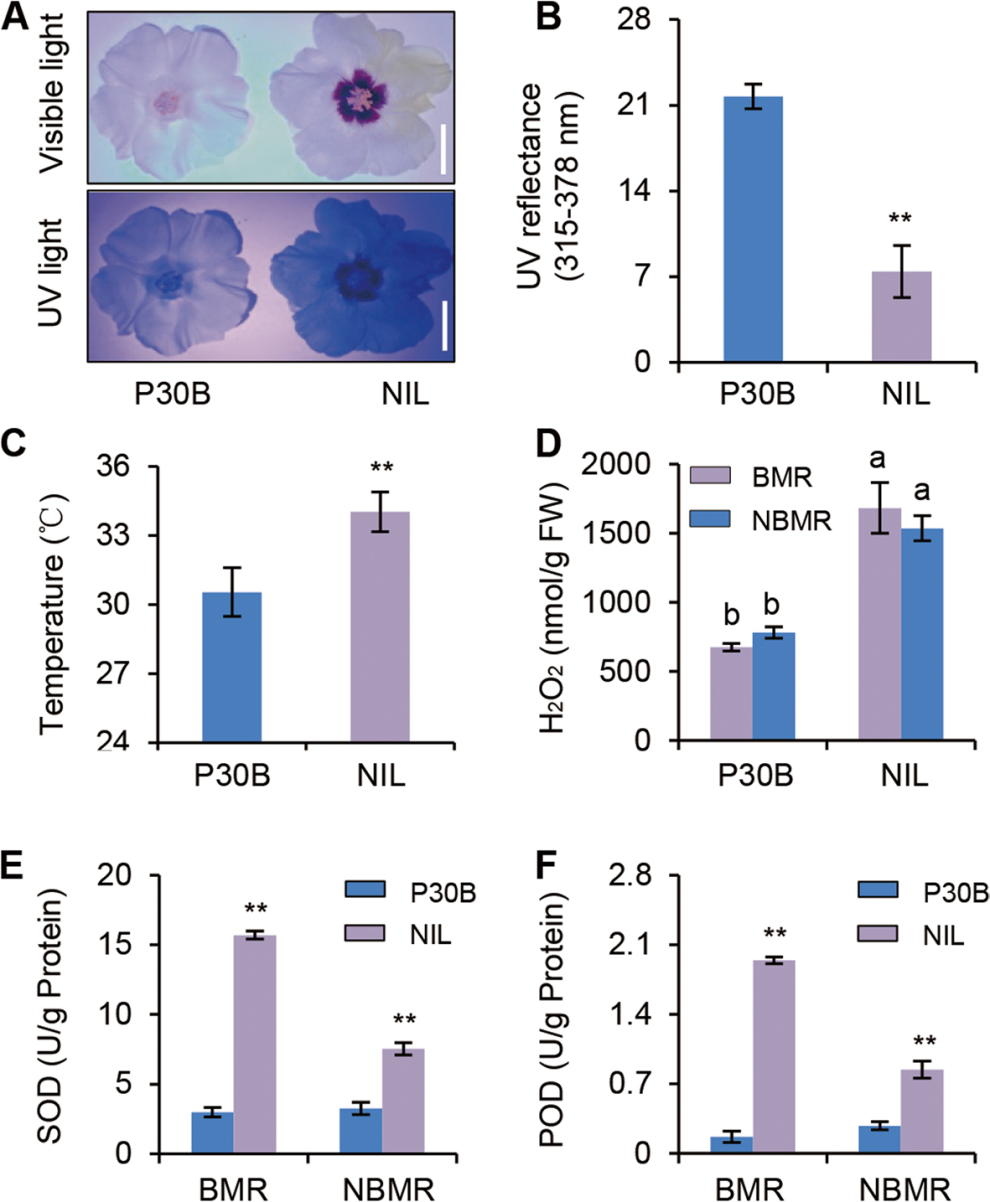
Measurement of H_2_O_2_ and antioxidant contents from floral petals of P30B and NIL. **A** Appearance of the flowers of P30B and NIL in visible light (upper) and UV light (lower). Scale bar, 2 cm. **B** UV reflectance in the flowers of P30B and NIL. Value is mean S.D. (*n* = 10, *P* ≤ 0.05, one-way ANOVA, Tukey’s HSD test). **C** Flowers petal temperature of P30B and NIL. Value is mean S.D. (*n* = 10). **D** Total extractable flower H_2_O_2_ contents in P30B and NIL. Value is mean S.D. (*n* = 3). **E**–**F** SOD activities and POD activities in the P30B and NIL flowers of beauty marker region (BMR) and non-beauty marker region (NBMR). Values are mean S.D. (*n* = 3). *P ≤ 0.05, **P ≤ 0.01. Student *t*-test was used to generate *P* value

We next assessed the UV reflectance in the petals of *G. hirsutum* P30B and *G. hirsutum* × *G. barbadense* near-isogenic lines (NILs). There were differences in the floral UV patterns but not in visible light reflectance. Moreover, NIL flowers exposed to UV light appeared significantly darker in color compared to P30B petals (Fig. 3A). Consistently, the NIL flowers showed comparatively lower levels of UV reflectance compared to P30B flowers (Fig. 3B). Altered flavonol contents and corresponding differences in UV patterns may impact physical traits (e.g., temperature) and/or physiological traits (e.g., ROS levels) in these plants (Fig. 3C, D). H_2_O_2_ levels were measured in the petals by quantifying DAB staining; this showed that NILs containing the beauty mark had considerably higher H_2_O_2_ levels compared to *G. hirsutum* (Fig. 3D). These data further supported the hypotheses that beauty marks and UV floral patterns were associated phenotypes, that UV exposure increased ROS generation in floral tissues, and that plants increased anthocyanin biosynthesis to mitigate the damaging effects of higher ROS levels.

We therefore evaluated the activities of two ROS-scavenging enzymes, superoxide dismutase (SOD) and peroxidase (POD), in P30B and NILs with and without the beauty mark. We found that SOD and POD activities were significantly increased in NIL petals compared to P30B petals (Fig. 3E, F). Moreover, SOD and POD activities were significantly higher in the beauty mark region than in the non-beauty mark region of NIL petals. These results demonstrated that the beauty mark protein GbBM contributed to ROS scavenging in *G. barbadense* and wild cotton plant flowers containing the beauty mark.

### Natural variation in the *Beauty Mark* CDS affected differences in expression between *G. barbadense* and *G. hirsutum*

To determine whether SNPs in *Beauty Mark* were associated with the beauty mark in *G. barbadense* and other wild cotton species, we retrieved the CDSs of *Beauty Mark* from 336 *G. barbadense* [43] and 419 *G. hirsutum* accessions [44]. Nucleotide sequence alignment revealed no change in the *Beauty Mark* sequence in the 336 *G. barbadense* accessions. However, comparison of *Beauty Mark* between *G. barbadense* and *G. hirsutum* revealed four synonymous polymorphisms (SNP 1, 14C ˃ A; SNP 2, 490 T ˃ C; SNP 3, 494C ˃ T; and SNP 4, 696A ˃ T) and one missense polymorphism (SNP 5, 510C ˃ T). SNP5 was associated with the beauty mark phenotype; all accessions bearing the beauty mark contained the T allele, whereas accessions without the beauty mark contained the C allele. This suggested that SNP5 played an important role in controlling petal spot development.

**Fig. 4:**
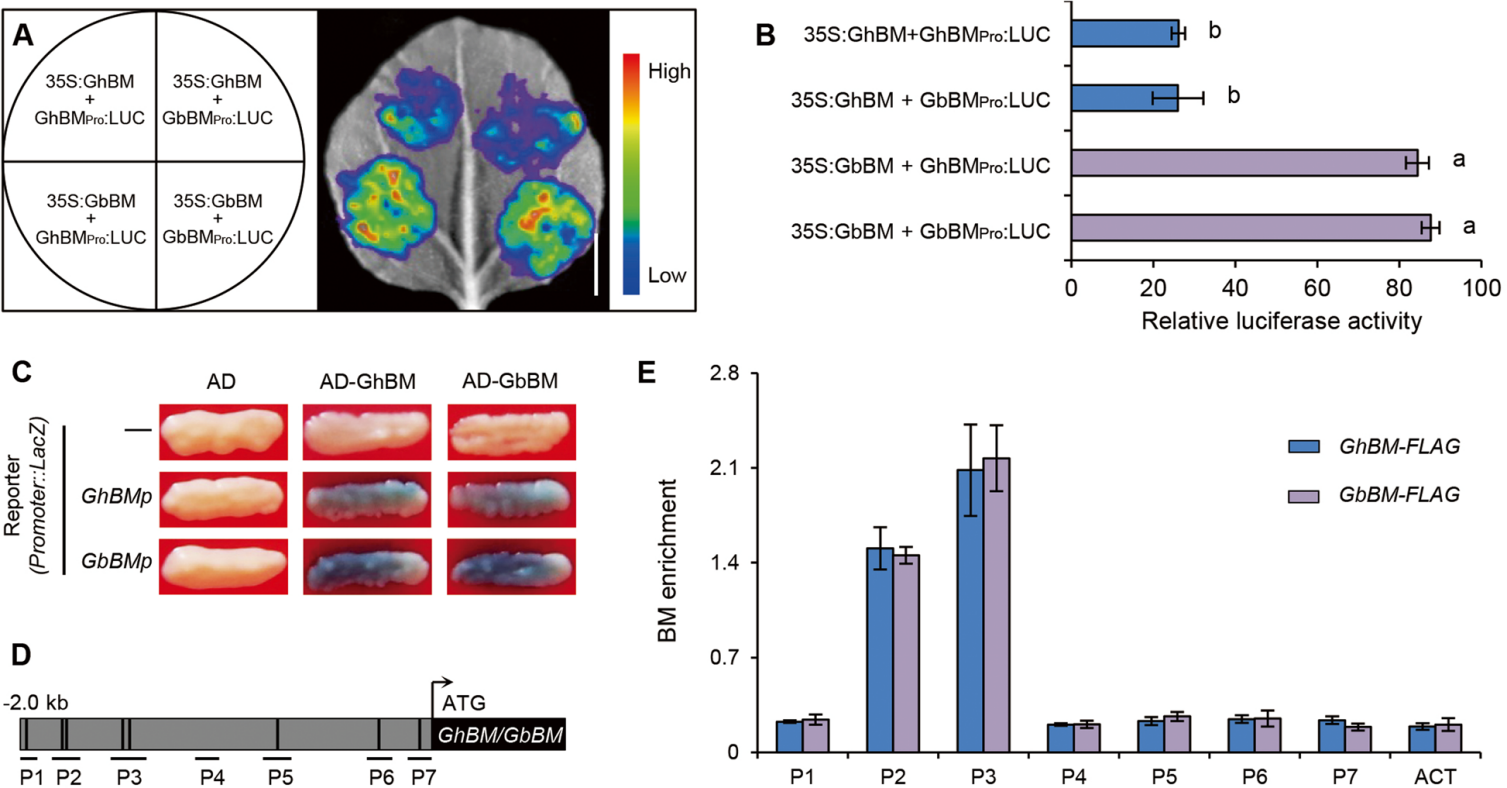
GhBM/GbBM directly targeting its own promoter. **A**, **B** Transient transcriptional activity analysis of Beauty Mark using luciferase transient expression. Transient expression assays showing that GhBM repress the transcriptional activity of both *GhBM*_*Pro*_*:LUC* and *GbBM*_*Pro*_*:LUC* (**A**). Representative images of *N. benthamiana* leaves 72 h after infiltration are shown. The left panel indicates the infiltrated constructs. Scale bar = 0.5 cm. Quantitative analysis of luminescence intensity with different letters indicates significant differences (*P* ≤ 0.05, one-way ANOVA, Tukey’s HSD test) (**B**). **C** AD-BM activates the expression of the *LacZ* reporter genes driven by the promoters of respective *GhBM* and *GbBM* in yeast. Representative data are shown from one of three biological replicates, which yielded similar results. **D** The regions tested by ChIP assays are shown in the schematic representation. The putative MYB-core elements in the promoter of *GhBM/GbBM* gene are indicated by black lines. **E** ChIP assays indicating the association of GbBM/GhBM with several regions in the promoters of GbBM and GhBM. The promoter of *GbActin1* was used for normalization. Bars represent means SD of three biological replicates. Student* t*-test was used to generate *P* value

We hypothesized that differences in *Beauty Mark* expression between *G. barbadense* and *G. hirsutum* could be caused by SNP5 in the CDS. We therefore conducted transient expression assays in *N. benthamiana* using the *GbBM* and *GhBM* promoters fused to the LUC reporter gene and *GbBM* and *GhBM* driven by the *35S* promoter. The results showed that luminescence intensity was repressed in both *GbBM* and *GhBM* promoter*-*driven reporters that expressed the *GhBM* allele, whereas co-expression of *GbBM* with either *GhBM*_Promoter_*-*LUC or *GbBM*_Promoter_-LUC showed high levels of luminescence (Fig. 4A, B). Next a yeast one-hybrid assay revealed that the GAL4 transcriptional activation domain-GbBM (AD-GbBM) and GhBM (AD-GhBM) fusion protein activates the *LacZ* reporter gene driven by the promoters both of *GbBM* and *GhBM*, respectively (Fig. 4C). Furthermore, chromatin immunoprecipitation with quantitative PCR (ChIP-PCR) assay confirmed that both GbBM and GhBM bound to the promoters fragments of *GbBM* and *GhBM* containing the MYB core motifs in the chromatin samples (Fig. 4D, E). It has been demonstrated that several R2R3 MYBs' activity is subject to an auto-regulatory mechanism, with the resultant effect occasionally being repressive. As a component of a negative auto-regulatory loop, *Arabidopsis* MYB4, whose encoded protein has the ability to bind its own promoter, inhibits transcription [45]. Petunia MYB27 [16], rice OsMYB4 [46], and *Arabidopsis* AtMYBL2 all exhibit a similar repressive mechanism [16, 47]. Taken together, these data demonstrated that polymorphisms in the CDS could be responsible for the lack of a beauty mark in *G. hirsutum.* Overall, these results demonstrated that SNP5 was directly associated with the petal spot phenotype in *G. barbadense*.

### SNP5 arose during *G. hirsutum* selection

**Fig. 5:**
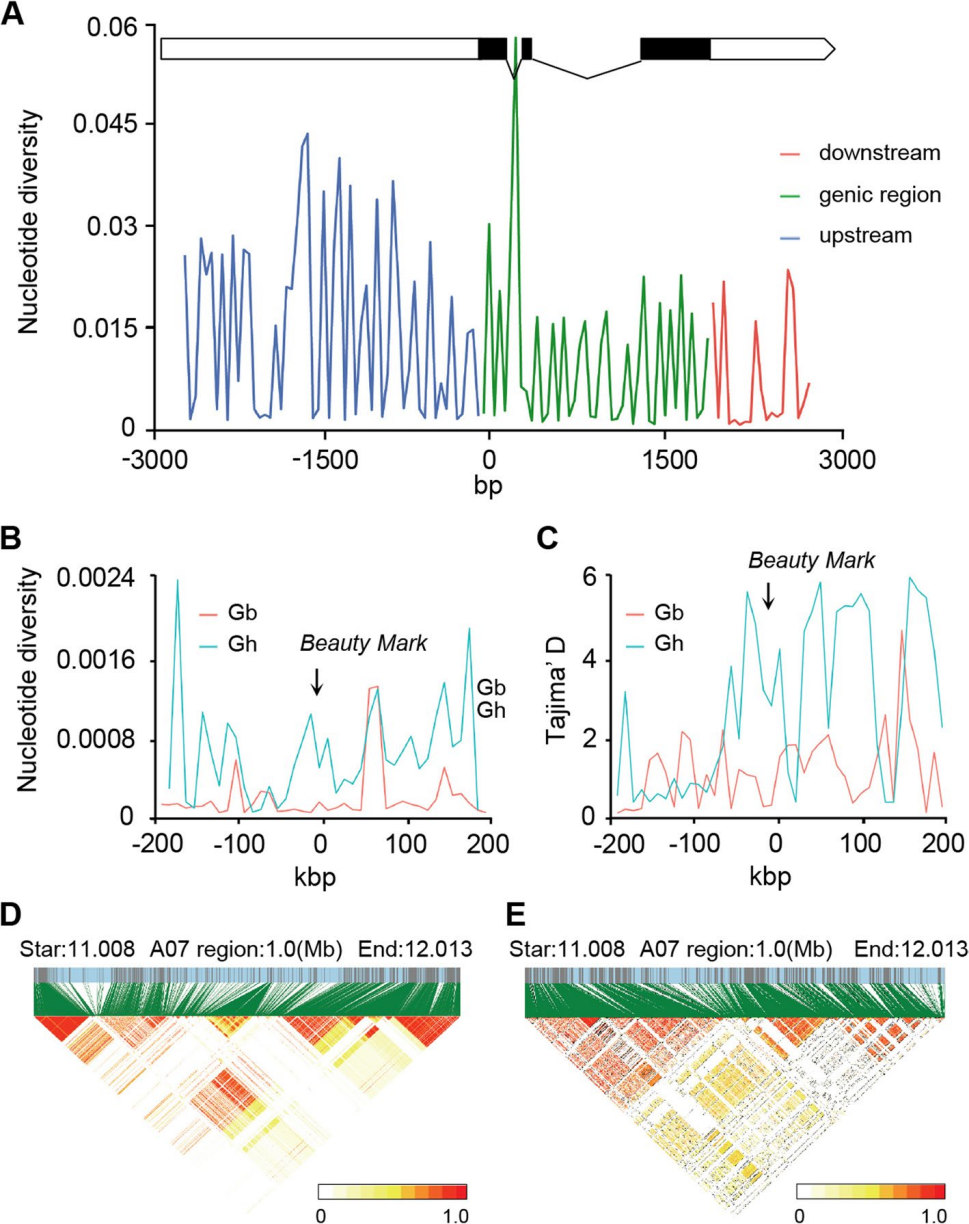
Nucleotide diversity and Tajima’ D test depict the selection pressure on GhBM during *G.hirsutum* domestication. **A** Nucleotide diversity in a fragment containing GhBM (including 3000 bp upstream, the coding region, and 3000 bp downstream). **B**, **C** To investigate whether the fragment containing GhBM was subject to selection pressure, nucleotide diversity (**B**) and Tajima’s *D* test (**C**) and were analyzed based on the total number of polymorphic sites in this fragment (including 200 kb upstream, the coding region, and 200 kb downstream). Gb,* G. barbadense.* Gh, *G. hirsutum*. **D**, **E** Comparison of linkage disequilibrium in the 1-Mb region around *Beauty Mark* between *G. barbadense* (**D**) and *G. hirsutum* (**E**)

To determine whether the nucleotide diversity in *G. hirsutum* was caused by selection pressure, we conducted Tajima’s *D* test on *GhBM* sequences. The value was significant for *G. hirsutum* (Tajima’s *D* = 3.599), indicating strong selection on the *GhBM* locus during *G. hirsutum* domestication (Fig. 5A and Table S1 and S2). To exclude the potential impact of geography on *GhBM* diversity, we further examined nucleotide diversity and Tajima’s *D*-values in the 400-kb region surrounding the *GhBM* locus and in *GbBM* in the 419 and 336 *G. barbadense* and *G. hirsutum* diversity panels, respectively, because selection may have led to a selective sweep in the flanking region (Fig. 5B, C). The linkage disequilibrium of the 1-Mb fragment containing *Beauty Mark* was comparatively greater in *G. hirsutum* than in *G. barbadense* (Fig. 5D, E). Taken together, these data showed that the T allele at SNP5 of *GhBM* in *G. hirsutum* was associated with the non-beauty mark phenotype in *G. hirsutum* cultivars, whereas the *C* at that position may be an ancient allele. A common evolutionary trend in plants (even in highly selfing plants) is “selfing syndrome,” in which traits associated with pollinator attraction (floral pigmentation) are lost or greatly reduced. However, it is unclear at present whether such trait reductions are favored by natural selection or result from reduced purifying selection coupled with genetic drift.

### *Beauty Mark* contributed to UV intensity-based geographical adaptation of *Gossypium* and modern cotton cultivars

**Fig. 6:**
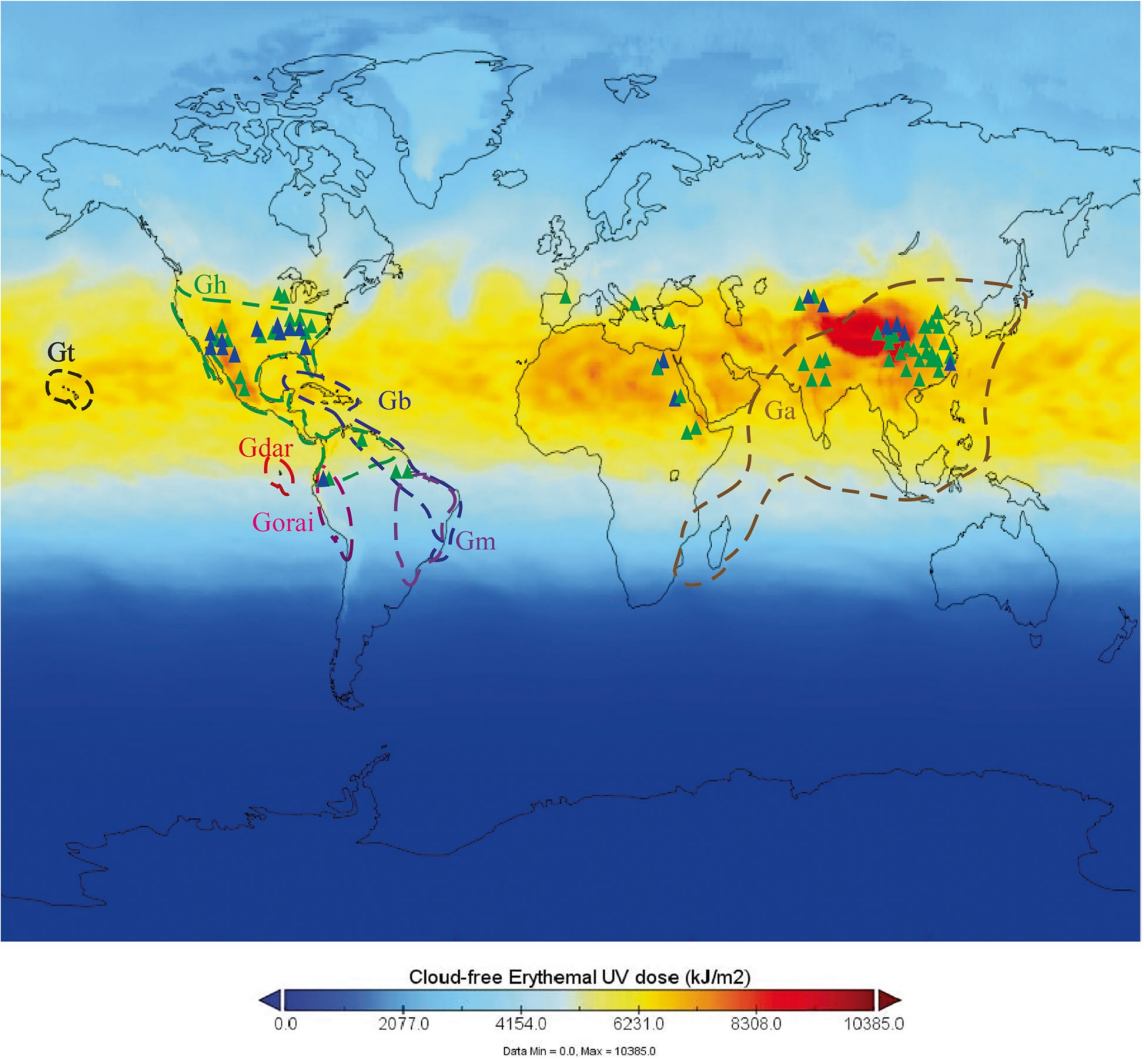
Geographic distribution of wild and cultivated *Gossypium* species and solar UV radiation levels (2002–2022). Ga, *G. arboretum*. Gh, *G. hirsutum*. Gt, *G. tomentosum*. Gb, *G. barbadense*. Gdar, *G. darwinii*. Gorai, *G. raimondii*. Gm, *G. mustelinum*. Blue triangles represent accessions from *G. barbadense* and green triangles represent accessions from *G. hirsutum*

Many *Gossypium* species are taxonomically well understood due to extensive molecular phylogenetic studies [48, 49]. To elucidate the role of the petal spot in wild cotton species and *G. barbadense* and the absence of the petal spot in *G. hirsutism* cultivars, we studied the geographic distribution of wild and cultivated cotton species under different UV exposure conditions. Geographically, *Gossypium* species (genomes A-G and K) were historically distributed across Northwestern, Central, and Western Australia; South and East Africa; Southeast Asia; the Galapagos Islands; Cape Verde Island; Peru; Mexico; and Southern Arizona. In contrast, accessions containing the AD genome (New World cotton) were geographically distributed across Hawaii, Brazil, the Dominican Republic, and the Galapagos Islands [50]. To examine the potential impact of geographical variation in UV radiation on the development of anthocyanin pigments, we used solar UV data (Erythema UV index) from the Tropospheric Emission Monitoring Internet Service for 2002 to 2022 (Fig. 6). The data showed that most of the regions in which cotton is economically important received the highest solar UV radiation levels during the cotton growth season, with UV index values ranging from 12 to 18. In addition, maximum UV index values were recorded in Northwestern, Central, and Western Australia; South and East Africa; Southeast Asia; the Galapagos Islands; Cape Verde Island; Peru; Mexico; and Southern Arizona. It is therefore likely that modern *G. hirsutum* cultivars, which are widely distributed across the globe, lack the beauty mark phenotype due to differences in petal UV patterns compared to *G. barbadense* and other wild cotton species that contain the beauty mark. Solar UV radiation may have led to increased anthocyanin pigmentation in cotton during its evolution. Collectively, our results suggest that environments with low latitude, high temperature, high solar UV exposure, and low precipitation may select (either directly or indirectly) for increased petal anthocyanin production, larger UV floral patterns, lower mean UV petal reflectance, and/or smaller flowers. UV floral patterns appear to confer a direct fitness advantage to individuals in high-UV environments by protecting the developing pollen and reproductive tissues from abiotic stress via increased anthocyanin biosynthesis.

## Discussion

Plants have multiple methods to balance solar energy capture with tolerance mechanisms (which regulate energy consumption, dissipation, and distribution), repair mechanisms, and avoidance mechanisms (which decrease light absorbance to prevent oxidative damage) [12, 51, 52]. Anthocyanins can significantly modify both the quantity and quality of incident light [53, 54] and absorb ultraviolet, blue, and green light [55]. The absorbance of UV-blue light by anthocyanins reduces the amount of light available to other light-absorbing organelles and protects the plant [56–58].

The cotton species *G. barbadense* is characterized by a petal spot (the beauty mark) at the base of each petal, which is caused by a group of anthocyanin-pigmented cells. The cultivated upland cotton genotypes are devoid of this petal spot. We previously identified *Beauty Mark*, a gene on chromosome A07 that encodes an R2R3-type GbMYB113 transcription factor, as a controller of petal spot development in *G. barbadense* [41]*.* In comparing the genomic sequences of MYB113 between several cultivated and wild cotton species, we identified five SNPs in the CDS; SNP5 (C/T) emerged as a candidate cause of petal spot absence in *G. hirsutum*. Furthermore, we hypothesized that increased UV floral patterns could result in higher temperatures within floral tissues. This phenomenon was clearly observed in the petals of NIL flowers, and led to elevated ROS production and increases in antioxidant activities. Because the beauty mark is located at the base of each petal, we postulated that *GbMB* expression may also serve to protect the nearby ovary and young reproductive structures through enhanced antioxidant activity. This hypothesis was supported by significantly higher SOD and POD activity in flower petals in NILs compared to *G. hirsutum*. This mechanism may also partially explain the growth of *G. barbadense* in geographic regions with high temperatures, high UV intensity, and a long frost-free period. Further, we established the mechanism by which petal spots protect the reproductive tissues of flowers from UV light and the evolutionary role of geographic distribution in the beauty mark phenotypes of wild species and New World cotton; this may be the basis for the apparent evolutionary convergence of red non-photosynthetic pigments, i.e., the beauty mark. We have established a model illustrating that UV-rich environments induce the accumulation of anthocyanin pigmentation in UV-absorbing plant organs to reduce the availability of UV light to reproductive tissues. However, further experiments are required to support this hypothesized protective function of anthocyanins for floral reproductive tissues.

Petal spots exist in ancestral cotton landraces but are absent in most cultivated cotton varieties. Few reports are available that positively correlate the petal spot with fiber yield and quality, indicating that human selection did not cause the non-spotted petal phenotype in *G. hirsutum* [40]. The reasons for the negative selection of anthocyanin biosynthesis in purple rice and cotton remain unclear [59]. Regulation of anthocyanin and flavonoid biosynthesis is related to plant adaptability to environments with a high incidence of solar UV exposure. Cotton species with responses to UV light that result in higher temperatures require more anthocyanin biosynthesis to scavenge ROS due to the temperature stress. *G. hirsutum* cultivars showed significant differences in UV patterning and consequently did not require high anthocyanin levels in the floral reproductive tissues and therefore have a broad geographic distribution. Our findings provide novel insights into the contributions of cotton petal spots to the geographic distribution and adaptation of cotton species.

## Conclusions

In conclusion, we found that the co-occurrence of the beauty mark and UV patterning led to significant increases in petal temperature, which in turn led to higher ROS levels. Furthermore, we demonstrated that there are differences between cotton species in absorbance and reflectance of UV light and that there are consequently variations in anthocyanin biosynthesis in floral tissues to scavenge ROS; these differences were associated with the geographic distribution of cotton species.

## Methods

### Plant materials and cultivation conditions

Two cultivated cotton species, *G. barbadense* and *G. hirsutum*, and nine wild cotton species (*G. herbaceum*, *G. capitis-viridis*, *G. anomalum*, *G. davidsonii*, *Rozi*, *G. klotzschianum*, *G. stocksii*, *G. bickii*, and *G. tomentosum*) were used in this study. Flowers were collected from wild cotton species at the National Wild Cotton Nursery in Hainan, China. Near-isogenic lines (NILs) were developed by backcrossing the *G. barbadense* line HaiR with the *G. hirsutum* line P30B eight times. UV light, ROS, and antioxidant data were collected from *G. hirsutum* P30B and NILs. Cotton plants used for temperature analyses and evaluation of UV light reflectance (described below) were collected from the experimental station at the Biotechnology Research Institute (BRI) of the Chinese Academy of Agricultural Sciences (CAAS) in Sanya (18°15′ N, 109° 30′ E). There were 30 cm between plants in each row and 90 cm between rows.

### *BM* sequence analysis

Sequence fragments of ~ 7 kb (from 2 kb upstream to 500 bp downstream of the CDS), as well 400 kb (± 200 kb) region around *BM* was selected for sequence analysis in 336 *G. barbadense* and 419 *G. hirsutum* accessions (Table S2) [43, 44, 60]. SNPs present in this region were extracted using the vcftools from vcf files for *G. hirsutum* and *G. barbadense* [61]. Tajima’s *D* and nucleotide diversity analysis were conducted using vcftools. In additionally, linkage disequilibrium analysis was conducted for 400 kb region of *BM* using the LD block show [60]. We then analyzed the results to identify patterns and differences in Tajima’s *D*, nucleotide diversity, and linkage disequilibrium between the *G. barbadense* and *G. hirsutum* accessions. The results of nucleotide diversity and Tajima’s D were plotted using ggplot2 [62]. We then sequenced *BM* in wild cotton species with the beauty mark (*G. herbaceum*, *G. capitis-viridis*, *G. anomalum*, *G. davidsonii*, *Rozi*, *G. klotzschianum*, *G. stocksii*, and *G. bickii*) and one species without it (*G. tomentosum*).

### Transient luciferase expression assay

The *GhBM* and *GbBM* promoter sequences were amplified from P30B and HaiR, respectively, and cloned into the pGreenII 0800-LUC reporter vector to drive firefly luciferase (*LUC*) expression. The *Renilla* luciferase (*REN*) gene, under the control of the cauliflower mosaic virus 35S promoter in the pGreenII 0800-LUC vector was used as the internal control. The CDSs of *GhBM* and *GbBM* were also cloned into the p2GW7 vector under the control of the 35S promoter as effectors. *Agrobacterium tumefaciens* strain GV3101 was transformed with each of the reporter and effector plasmids; the transformed lines were then co-infiltrated into *N. benthamiana* leaves. The NightSHADE LB 985 imaging system (Berthold) was used to assess LUC activity 72 h after infiltration.

### Yeast one-hybrid assay

Yeast one-hybrid experiment was carried out according to the method described previously [41]. To generate AD-GbBM and AD-GhBM, the full-length GbBM and GhBM were amplified and cloned into the vector pJG4-5 vector (Clontech). To generate *GbBMp::LacZ* and *GhBMp::LacZ* reporter constructs, the promoter fragments were amplified and then separately subcloned into the pLacZi2μ vector. Primers were listed in Table S3. Transformants were cultivated on proper drop-out plates containing *X*-gal for blue color show. Representative data was shown from one of the three biological replicates which yield similar results.

### Chromatin immunoprecipitation assays

The *G. hirsutum* line R15 and *G. barbadense* line HaiR and their transgenic calli containing the *35S:GbBM-FLAG* and *35S:GhBM-FLAG* constructs were used for ChIP experiment as described previously [41]. About 2 g of each calli were collected and cross-linked in 1% formaldehyde for 15 min, next neutralized with 0.125 M of glycine for 5 min. After washing with distilled water five times, the samples were ground to power in liquid nitrogen. The chromatin complexes were isolated and sonicated, and then the chromatin complex was immunoprecipitated by anti-FLAG antibody (Abmart, catalog number M20008). The precipitated DNA was analyzed with quantitative real-time PCR. Primers used for ChIP-PCR are listed in Table S3. The promoter of *Actin1* was used as negative control. Three independent biological repeats were performed.

### H_2_O_2_ measurements

H_2_O_2_ was extracted from fully opened flowers during peak sunlight hours, as previously described [63]. After extraction, H_2_O_2_ was quantified using the Hydrogen Peroxidase Assay Kit (Solarbio, China) following the manufacturer’s instructions. A mass spectrometer was used to measure absorbance at 415 nm.

### DAB staining

The in situ detection of hydrogen peroxide was carried out in fully opened flower petals of cultivated and wild cotton species by staining with DAB using the procedure described by [64].

### Determination of SOD and POD activities

SOD and POD activities were measured as previously described [63]. Total protein content was measured using a Bradford protein assay kit (Sangon Biotech, Shanghai, China).

### Temperature measurements and UV reflectance scoring

Flower images were captured with a Panasonic DMC-LX5GK 24-mm wide camera. UV light was provided by a 253.7 nm UV-emitting lamp. Flowers were directly compared and scored for specific UV patterns. LightScout UV meters (3414F) and LightScout UV sensors (3637I) (Spectrum Technologies USA) with a capacity of 250–400 nm were used to measure UV radiation in an open field with a range of 0–200 μMol/m^2^/s (± 5%). Three UV sensors were installed at the field research station of the BRI. UV sensor data was collected using WatchDog 2000 series data loggers and analyzed with Spec Pro 9 (Spectrum Technologies USA). Flower temperatures were recorded with a temperature sensor gun (Thermo Fisher Scientific) following the manufacturer’s instructions. Temperatures were consistently measured on sunny days during the same light period.

## Supplementary Information

**Additional file 1:** **Table S1.** Tajima’s D test for *GhBM* and *GbBM* sequences. **Table S2.** Cotton accessions used for sequence analysis. **Table S3.** Sequences of primers used in this study.

**Additional file 2.** Includes individual values for quantitative data depicted in Figs. 1, 3 and 4.

## Data Availability

All data generated or analyzed during this study are included in this published article, its supplementary information files, and publicly available repositories. Quantitative data generated in this study are presented in Additional file 2.

## Abbreviations

bHLH: Basic helix-loop-helix
BM: Beauty mark
CDS: Coding sequence
DAB: 3,3′-Diaminobenzidine
LUC: Firefly luciferase
MBW: MYB–bHLH–WD repeat protein
MYB: V-myb avian myeloblastosis viral oncogene homolog
NILs: Near-isogenic lines
ROS: Reactive oxygen species
qRT PCR: Quantitative reverse transcription polymerase chain reaction
POD: Peroxidase
REN: *Renilla luciferase*
SNP: Single nucleotide polymorphism
SOD: Superoxide dismutase
UV: Ultraviolet
